# Identification of Novel Hemangioblast Genes in the Early Chick Embryo

**DOI:** 10.3390/cells7020009

**Published:** 2018-01-31

**Authors:** José Serrado Marques, Vera Teixeira, António Jacinto, Ana Teresa Tavares

**Affiliations:** 1 CEDOC, Chronic Diseases Research Centre, NOVA Medical School, Universidade NOVA de Lisboa, Campo dos Mártires da Pátria, 130, 1169-056 Lisbon, Portugal; jose.marques@nms.unl.pt (J.S.M.); antonio.jacinto@nms.unl.pt (A.J.); 2 Instituto Gulbenkian de Ciência, Rua da Quinta Grande, 6, 2780-156 Oeiras, Portugal; teixeira@igc.gulbenkian.pt

**Keywords:** chicken embryo, yolk sac, hemangioblast, microarray analysis, novel genes

## Abstract

During early vertebrate embryogenesis, both hematopoietic and endothelial lineages derive from a common progenitor known as the hemangioblast. Hemangioblasts derive from mesodermal cells that migrate from the posterior primitive streak into the extraembryonic yolk sac. In addition to primitive hematopoietic cells, recent evidence revealed that yolk sac hemangioblasts also give rise to tissue-resident macrophages and to definitive hematopoietic stem/progenitor cells. In our previous work, we used a novel hemangioblast-specific reporter to isolate the population of chick yolk sac hemangioblasts and characterize its gene expression profile using microarrays. Here we report the microarray profile analysis and the identification of upregulated genes not yet described in hemangioblasts. These include the solute carrier transporters *SLC15A1* and *SCL32A1*, the cytoskeletal protein *RhoGap6*, the serine protease *CTSG*, the transmembrane receptor *MRC1*, the transcription factors *LHX8*, *CITED4* and *PITX1*, and the previously uncharacterized gene *DIA1R*. Expression analysis by in situ hybridization showed that chick *DIA1R* is expressed not only in yolk sac hemangioblasts but also in particular intraembryonic populations of hemogenic endothelial cells, suggesting a potential role in the hemangioblast-derived hemogenic lineage. Future research into the function of these newly identified genes may reveal novel important regulators of hemangioblast development.

## 1. Introduction

During vertebrate embryogenesis, there is a close developmental relationship between hematopoiesis and vasculogenesis. In the early embryo, the first hematopoietic and endothelial cells arise in the extraembryonic yolk sac blood islands from a common precursor known as the hemangioblast [1,2]. Recent evidence suggests that hemangioblasts give rise to hematopoietic cells through two types of intermediate progenitors, hemogenic angioblasts and hemogenic endothelial cells [3,4]. In the yolk sac, a first wave of hematopoiesis arises from hemogenic angioblasts that give rise to primitive hematopoietic cells, such as primitive erythrocytes, embryonic macrophages and megakaryocytes [4,5,6]. Also in the yolk sac, a second wave of hematopoiesis originates from hemogenic endothelial cells that give rise to definitive erythrocytes and to most myeloid lineages, including tissue-resident macrophages and microglial cells that persist into adulthood [7,8]. Finally, a third wave of hematopoiesis arises from intraembryonic hemogenic endothelial cells and produces definitive hematopoietic stem/progenitor cells (HSPC) that will colonize the fetal hematopoietic organs [9]. Cell-tracing studies have shown that these intraembryonic precursors also have an extraembryonic origin, as they migrate from the yolk sac prior to the onset of circulation [10,11]. Together, these evidences suggest that most (if not all) hematopoietic cells in the embryo derive from yolk sac hemangioblasts.

The identification of novel hemangioblast markers and regulators has great clinical potential in regenerative medicine, for it may contribute to the implementation of new hemangioblast-based therapies for the treatment of various hematologic and vascular disorders. Although several factors have been shown to play a role in hemangioblast formation, such as Lmo2 [12,13], Tal1/Scl [14], Runx1 [15] and Sox7 [16], our knowledge on the molecular players involved in hemangioblast specification and differentiation remains largely incomplete. We therefore sought to identify novel potential hemangioblast regulators by analyzing the gene expression profile of yolk sac hemangioblasts isolated from the early chick embryo, as previously attempted in other model systems [17,18]. In the past, we identified and characterized a novel hemangioblast-specific enhancer (Hb) that is able to specifically drive the expression of a reporter gene (enhanced green fluorescent protein, eGFP) in yolk sac hemangioblasts of the chicken embryo [19,20]. This work introduced the Hb-eGFP reporter as a powerful tool for labeling the hemangioblast population and studying the dynamics of blood island morphogenesis in live imaging assays. Moreover, this reporter was used to describe the transcriptional profile of the hemangioblast [19]. In this communication, we report the pathway and gene network analysis of the hemangioblast transcriptome and the identification of novel genes expressed in yolk sac hemangioblasts. In addition to genes known to have a role in other cell types and developmental processes, we introduce a previously uncharacterized gene, *DIA1R*, and describe its expression pattern in the chick embryo at different stages of development.

## 2. Materials and Methods

### 2.1. Embryo Ex Ovo Electroporation

Fertilized chicken eggs were purchased from Quinta da Freiria (Bombarral, Portugal) and incubated for the appropriate period at 37.5 °C in a humidified incubator. Embryos were staged according to Hamburger and Hamilton (HH; [21]) and processed as previously described [19]. In brief, HH3 chicken embryos were injected with Hb-eGFP and pCAGGS-RFP reporter plasmids and electroporated using 2-mm square electrodes (CY700-1Y electrode; Nepa Gene, Chiba, Japan) and a square wave electroporator (ECM830; BTX, Holliston, MA, USA). Electroporated embryos were grown until stages HH5-6 in New culture [22] and imaged using a Zeiss SteREO Lumar.V12 fluorescence stereomicroscope equipped with a Zeiss MRc.Rev3 camera and ZEN 2 Pro software (Carl Zeiss, Oberkochen, Germany).

### 2.2. Immunohistochemistry

Hb-eGFP-electroporated embryos were fixed in 4% paraformaldehyde, cryoprotected in 15% sucrose, embedded in 7.5% gelatine/15% sucrose and cryosectioned at 20 μm. Immunostaining was performed with a primary antibody against the extracellular domain of avian VEGFR2 (gift from Anne Eichmann) [23] and a secondary antibody labeled with Alexa Fluor 568 (A11004; Thermo Fisher Scientific, Waltham, MA, USA). Images were acquired on a Leica DMRA2 upright microscope (Leica Microsystems, Wetzlar, Germany) with a CoolSNAP HQ CCD camera (Photometrics, Tucson, AZ, USA) and MetaMorph V7.5.1 software (Molecular Devices, Sunnyvale, CA, USA).

### 2.3. Microarray Data Analysis

Microarray expression profiling of the yolk-sac hemangioblast transcriptome is described in detail in our previous work [19]. In brief, embryos were electroporated with the Hb-eGFP and pCAGGS-RFP reporter constructs, harvested at stage HH5-6 and dissociated into a single cell suspension. The eGFP+/RFP+ and eGFP-/RFP+ cell populations were sorted on a Mo-Flo high-speed fluorescence-activated cell sorter (Beckman Coulter, Brea, CA, USA). Total RNA was isolated from triplicates of each population and processed for RNA integrity evaluation, reverse transcription and amplification. cRNA samples were hybridized against six Affymetrix GeneChip Chicken Genome arrays and scanned on an Affymetrix GeneChip scanner 3000 7G (Thermo Fisher Scientific). The microarray dataset was deposited in NCBI’s Gene Expression Omnibus (GEO) under the accession number GSE32494.

Differentially expressed genes with a fold change greater than 1.2 were analyzed using Ingenuity Pathway Analysis (IPA) software (Ingenuity Systems, Redwood City, CA, USA; www.ingenuity.com). The Functional Analysis was used to identify the biological processes and/or diseases, whereas the Canonical Pathways Analysis was used to identify the signaling pathways that were most significant to the dataset. The significance of the association between the dataset and the functional class or canonical pathway was expressed as negative log *p*-value using Fisher’s exact test. The molecular relationships between gene products were represented in a network generated from information contained in the Ingenuity Pathways Knowledge Base. This analysis was restricted to four functional classes associated with early embryonic development: Cellular Development, Cardiovascular System Development and Function, Organismal Development, Organ Development and Cell Signaling.

### 2.4. In Situ Hybridization

The chick *DIA1R* riboprobe was generated from a fragment of the cDNA clone ChEST746d11 (nucleotides 1–585; GenBank accession number BX931741). For whole-mount in situ hybridization, chicken embryos were collected at stages HH3 to HH18 and processed as previously described [24]. Selected embryos were dehydrated in 30% sucrose, embedded in gelatin, frozen and cryosectioned. Embryos at embryonic day 10 (E10) were cryosectioned before being processed for in situ hybridization on tissue sections, as described [25]. Whole-mount embryos were imaged on Zeiss SteREO Lumar.V12, whereas tissue sections were imaged on a Leica DMLB2 upright microscope, equipped with a Leica DFC250 color CCD camera (Leica Microsystems), using IrfanView software (Irfan Skiljan, Wiener Neustadt, Austria; www.irfanview.com).

## 3. Results and Discussion

**Figure 1:**
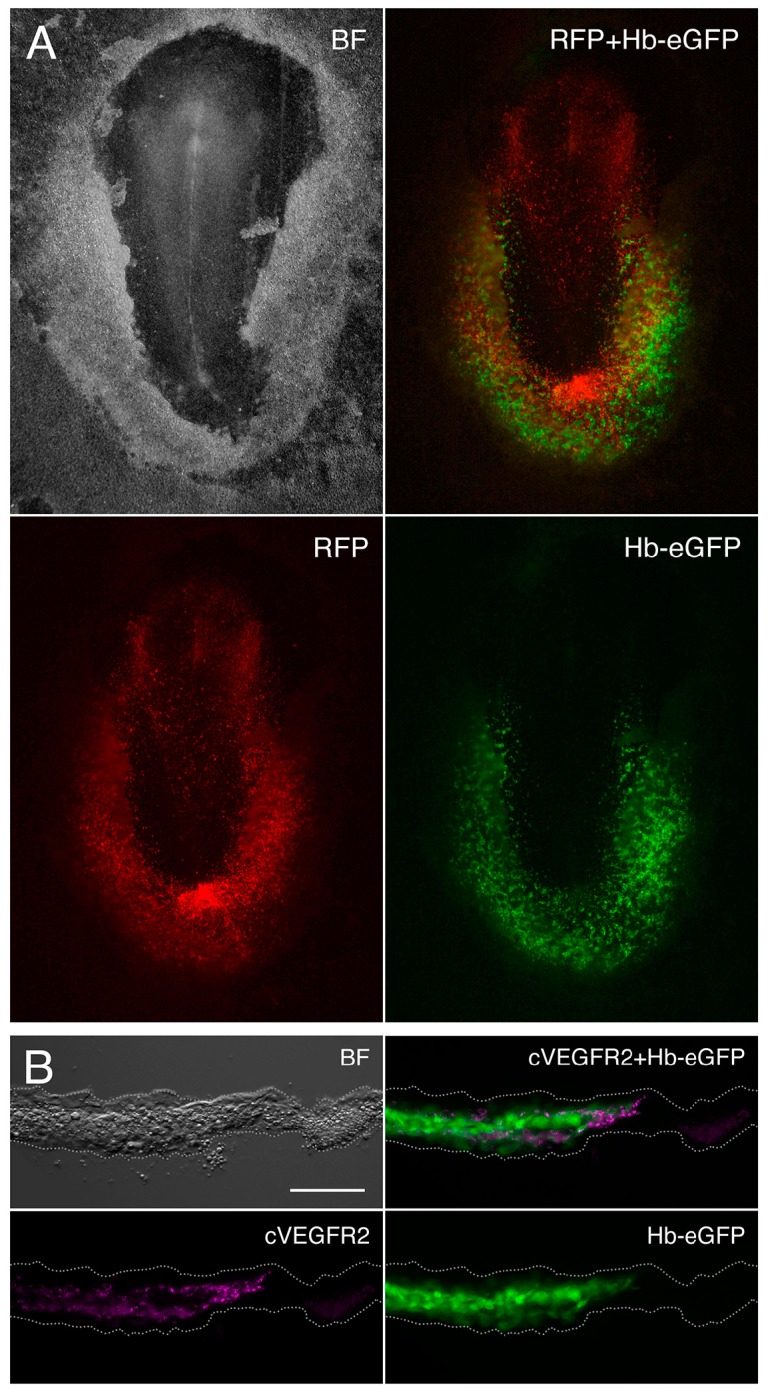
Expression of the yolk sac hemangioblast reporter in the early chick embryo (HH5). (**A**) Chick embryo co-electroporated with the pCAGGS-RFP ubiquitous reporter (red) and the Hb-eGFP hemangioblast reporter (green); (**B**) Transverse section of an Hb-eGFP-electroporated embryo immunolabeled for cVEGFR2 (magenta). At this early stage, the Hb-eGFP reporter specifically labels the yolk sac population of hemangioblasts, which can be identified by the expression of cVEGFR2 (**B**). This membrane receptor is detected at the surface of the eGFP-expressing cells. BF, bright field. Scale bar: 100 µm.

During the study of chick *Cerberus* transcriptional regulation [26], we isolated a *cis*-regulatory region that drives reporter gene expression specifically in yolk sac hemangioblasts [19]. The specificity of this hemangioblast reporter (Hb-eGFP) is highlighted in Figure 1. In chick embryos co-electroporated with Hb-eGFP and the ubiquitous reporter pCAGGS-RFP, eGFP fluorescence is restricted to a population of cells in the posterior extraembryonic region (Figure 1A) and co-localizes with cVEGFR2 (Flk1), a marker of early hemangioblasts (Figure 1B; [23]).

In our previous work, we used the Hb-eGFP reporter to isolate the hemangioblast population and characterize its gene expression profile by microarray analysis ([19]; GSE32494). At the time, this analysis was used to confirm the specificity of the hemangioblast reporter. Here we have taken a deeper look at our microarray data in order to uncover the pathways most active in the hemangioblast and identify novel genes expressed in this cell population.

### 3.1. Gene Expression Analysis of the Hemangioblast Transcriptome

**Figure 2:**
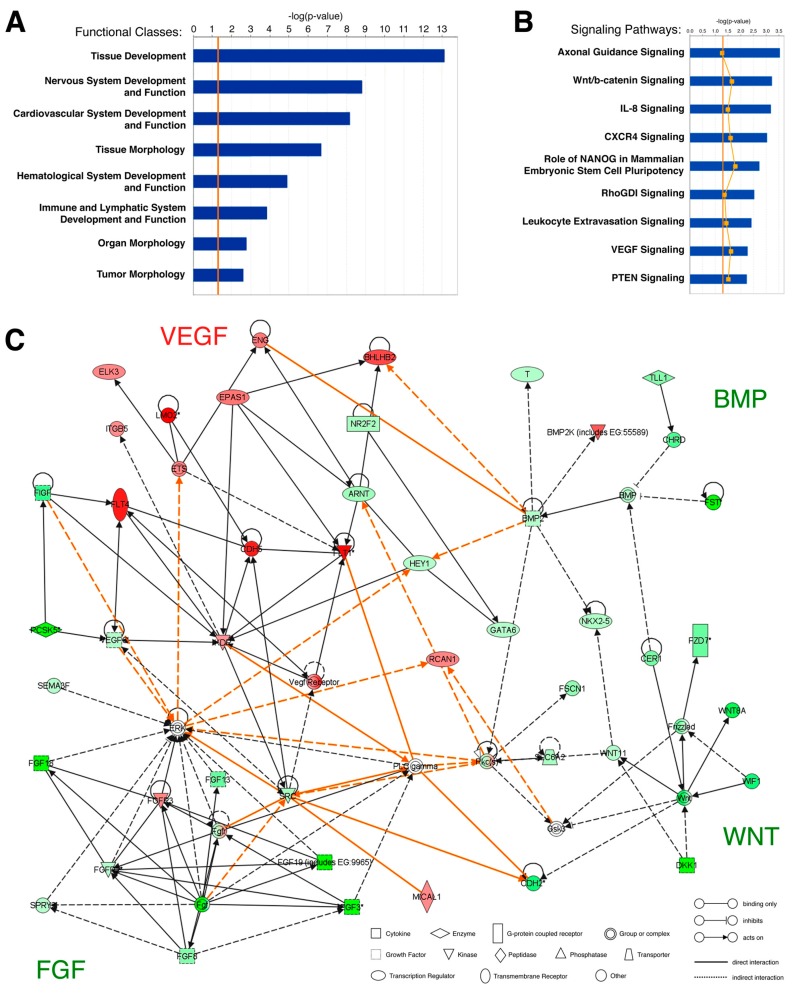
Ingenuity pathway analysis of genes differentially expressed in yolk sac hemangioblasts. (**A**) Top classes of biological functions and (**B**) canonical signaling pathways most significantly represented in the Hb-eGFP+ microarray dataset (Ingenuity Pathways Analysis (IPA) library; Ingenuity Systems, www.ingenuity.com). Genes that met the fold change cutoff of 1.2 were considered for the analysis. Bars indicate the minus log of the *p*-value of each functional class/canonical pathway. The threshold line (orange) corresponds to a *p*-value of 0.05. The yellow line in (**B**) represents the ratio between the number of genes from the dataset in a given pathway that meet the cutoff criteria and the total number of genes of that pathway; (**C**) Network diagram representing the molecular relationships between genes differentially expressed in hemangioblasts. This graphical representation generated by IPA includes gene products of four functional classes: Cellular Development, Cardiovascular System Development and Function, Organismal Development, Organ Development and Cell Signaling. Gene products are represented as nodes (shapes) and the biological relationship between two nodes is represented as an edge (line). Orange lines represent interactions between gene products from different canonical pathways. All edges are supported by at least one reference from the literature, from a textbook, or from canonical information stored in the Ingenuity Pathways Knowledge Base. The intensity of the node color indicates the degree of upregulation (red) or downregulation (green). Nodes are displayed using various shapes that represent the functional class of the gene product, while edges are displayed with various labels that describe the nature of the biological relationship between the nodes (see legend in the figure).

For the microarray analysis of the yolk-sac hemangioblast, we electroporated chick embryos with Hb-eGFP and pCAGGS-RFP reporter constructs, isolated the Hb-eGFP+/RFP+ and Hb-eGFP-/RFP+ cell populations and compared their gene expression profiles [19]. We analyzed the microarray dataset using Ingenuity Pathway Analysis (IPA; Ingenuity Systems, http://www.ingenuity.com) in order to identify the functional classes and signaling pathways that were most significantly represented in the hemangioblast transcriptome (Figure 2).

The functional pathway analysis identified 65 classes of biological functions that are significantly enriched in the dataset from hemangioblasts, eight of which are displayed in Figure 2A. As expected, hemangioblast genes were assigned to functional classes related to embryonic development, such as *Tissue Development* and *Organ Morphology*. In addition, the high representation of the classes *Cardiovascular System Development and Function*, *Hematological System Development and Function* and *Immune and Lymphatic System Development and Function* suggests that hemangioblasts express genes associated with both vascular and hematopoietic lineages, as previously shown [27].

The canonical pathway analysis identified 78 pathways that are significantly enriched in the dataset from hemangioblasts, nine of which are displayed in Figure 2B. Two of these pathways are the VEGF signaling pathway, which play a well-established role in vasculogenesis [28], and the CXCR4 signaling pathway, which regulates HSPC homing and engraftment in the bone marrow [29] and may be involved in the migration of yolk sac hemangioblast-derived angioblasts into intraembryonic regions [7,11]. Alternatively, CXCR4 signaling may modulate the hemogenic potential of the yolk sac hemangioblast, as recently shown in embryonic stem cell cultures [30]. We also identified several hemangioblast genes that are involved in axon guidance. Indeed, the vascular and neural networks are known to have several common morphogenetic signals [31]. Interestingly, another pathway over-represented in hemangioblasts is the leukocyte extravasation signaling pathway, which includes molecules responsible for the interactions between blood and endothelial cells [32]. These molecules are likely to play a similar role in the blood-endothelial interactions that take place during the differentiation of hemangioblasts in the blood islands.

We then designed and analyzed the network of molecular interactions of the major signaling pathways differentially expressed in hemangioblasts (Figure 2C). As expected, many upregulated genes belong to the VEGF signaling pathway, such as *FLT1* (*VEGFR1*) and *FLT4* (*VEGFR3*), or interact with it, such as *CDH5* (*VE-cadherin*), an endothelium adhesion molecule that is expressed in hemogenic endothelial cells prior to their differentiation [33], and *LMO2*, a transcription factor essential for hemangioblast development and hematopoiesis [12,13,34]. On the other hand, several downregulated genes belong to or interact with the BMP, FGF and WNT signaling pathways. These three pathways include genes that are highly expressed in cell types other than the hemangioblast, such as the paraxial mesoderm (e.g., *FST*—BMP signaling pathway; [35]), primitive streak (e.g., *FGF19*—FGF signaling pathway; [36]) and axial mesendoderm (e.g., *DKK1*—NT signaling pathway; [37]).

### 3.2. Identification of Novel Hemangioblast Genes

In addition to genes that are known to play a role in hemangioblast development, such as *LMO2* [12,13] and *TAL1/SCL* [14], our differential screening of hemangioblast transcripts led to the identification of several genes unknown to have a function in hematopoiesis or vasculogenesis, as well as some previously uncharacterized genes (Table 1). These include *SLC15A1* (+5.1) and *SCL32A1* (+4.19), *RhoGap6* (+3.07), *CTSG* (+3.7), *MRC1* (+3.2), *LHX8* (+2.77), *CITED4* (+2.23), *PITX1* (+2.2), and the novel gene *DIA1R* (+4.2).

SLC15A1 and SLC32A1 are members of the solute carrier family. The transmembrane transporter SLC15A1 is involved in amino acid uptake in the intestinal epithelium [38], whereas the vesicular transporter SLC32A1 acts as a carrier of inhibitory amino acid neurotransmitters in the central nervous system [39]. Their expression in hemangioblasts may indicate that these cells have a particular amino acid requirement. The cytoskeletal protein RhoGap6 was shown to promote the formation of filopodia-like processes in mammalian cell cultures [40], and it may have a similar role in hemangioblasts as they actively migrate in the extraembryonic region [19].

The serine protease CTSG (Cathepsin G) is expressed at the promyelocytic stage of myeloid development [41]. In addition, CTSG participates in tissue remodeling at sites of inflammation [42] and in the degradation of endothelial VE-cadherin during neutrophils transmigration [43]. In hemangioblasts, CTSG may play an active role in extracellular matrix remodeling during blood island formation and endothelial-to-hematopoietic transition. MRC1 is a transmembrane mannose receptor that mediates the phagocytosis of microorganisms by antigen-presenting cells [44]. During development, *MRC1* transcripts are found in the zebrafish caudal hematopoietic tissue and endothelial cell precursors [45] and in the mouse yolk sac blood islands [46]. Taken together, the presence of both *CTSG* and *MRC1* in yolk sac hemangioblasts indicates that myeloid lineage genes are already expressed in these progenitors.

LHX8 is involved in the differentiation of cholinergic neurons in the mouse telencephalon [47], CITED4 regulates the proliferation of embryonic stem cell-derived cardiac progenitor cells [48], and PITX1 plays a role in pituitary and hindlimb development [49]. These transcription factors may also regulate the differentiation of HSPCs, as do their respective family members LHX2 [50], CITED2 [51] and PITX2 [52]. In the future, the expression of these newly identified genes in yolk sac hemangioblasts should be validated in the early embryo. In addition, further investigation will be required to resolve their potential roles in the hemangioblast.

### 3.3. Expression Pattern of DIA1R in the Chick Embryo

The second most highly expressed gene in the hemangioblast transcriptome was the chick ortholog of human *cXorf36* or *DIA1R* (deleted in autism 1 related; +4.2 fold change; Table 1), a gene implicated in autism spectrum disorders and X-linked mental retardation [53,54]. The chick *DIA1R* gene (*cDIA1R* or *C1HXorf36*) encodes a protein of 430 amino acids that is 65% identical and 87% similar to the human protein [54]. *DIA1R* genes are found exclusively in vertebrates and their function is largely unknown. Based on sequence analysis, DIA1R proteins were predicted to contain a signal peptide and a highly conserved PIP49_C protein-kinase domain, characteristic of the FAM69 family of kinase-like proteins [53,55]. These features suggest that DIA1R proteins may regulate molecular traffic or interfere with the function of secreted factors [53].

**Figure 3:**
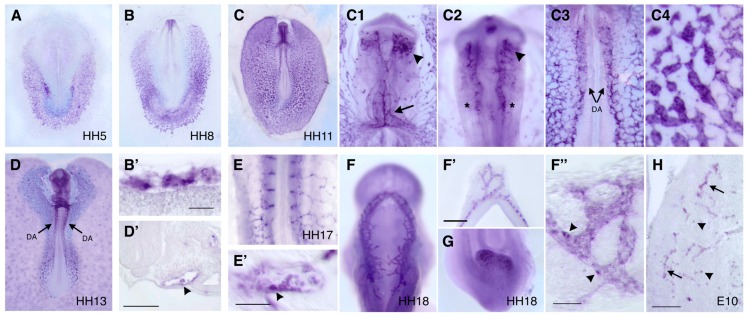
*cDIA1R* expression in the chick embryo. *cDIA1R* in situ hybridization was performed on whole-mount embryos at HH5 (**A**), 8 (**B**), 11 (**C**), 13 (**D**), 17 (**E**), 18 (**F**,**G**) and on a E10 brain cryosection (**H**). (**B’**,**D’**,**E’**,**F’**,**F”**) Sections of correspondent whole-mount embryos. (**C1**–**C4**) Regions of embryo in (**C**) at high magnification. *cDIA1R* expression starts to be detected at HH5 (**A**) in the extraembryonic mesoderm that will form the yolk sac blood islands at later stages (**B**,**B’**,**C4**). In HH11 embryos (**C**), *cDIA1R* is also expressed in the endocardium (arrow in **C1**), in the developing head vasculature (arrowheads in **C1** and **C2**), in cells associated with the dorsal mid- and hindbrain (asterisks in **C2**) and in the dorsal aorta region (DA; **C3**). At later stages (**D**–**H**), *cDIA1R* expression is detected in most blood vessels of the embryo, such as the dorsal aorta (DA in **D**,**D’**), intersomitic vessels (**E**), head vasculature (**E’**,**F**,**F’**,**F”**) and allantois (**G**). Higher intensity is found in particular blood vessel cells that resemble hemogenic endothelial cells (**D’**,**E’**,**F”**; arrowheads). In the brain neuroepithelium (**H**), *cDIA1R* is detected both in the neurovasculature (arrows) and in isolated cells that may be microglial cells (arrowheads). Scale bars: 50 μm in **B’**,**E’**,**F”**; 100 μm in **D’**,**F’**.

The analysis of *cDIA1R* expression during chick development revealed that this gene is expressed in yolk sac hemangioblasts, blood islands and endothelial cells of the dorsal aorta, endocardium and head vasculature (Figure 3), which are regions known to have hemogenic capability [56]. In particular, *cDIA1R* expression appears to be higher in cells that are morphologically similar to hemogenic endothelial cells (Figure 3D’,E’,F”). In the brain neuroepithelium, *cDIA1R* is expressed in the vascular endothelium and in isolated cells that resemble microglial cells (Figure 3H; [57]). Consistently with our observations, transcripts of *DIA1R* orthologs have been detected in hematopoietic progenitors and endothelial cells in zebrafish embryos ([17]; *cc058* gene), in endothelial and microglial cells from embryonic and adult mouse brains ([58]; *4930578C19Rik* gene) and in human endothelial cells [59]. Taken together, these findings suggest that DIA1R may be a good marker and potential regulator of the intraembryonic hemogenic lineages that derive from yolk sac hemangioblasts.

Over the past decade, increasing evidence supports a key role for microglia in the pathogenesis of neurodevelopmental disorders such as autism [60,61,62]. In addition, reduced rates of angiogenesis and perfusion abnormalities have been found in autistic brains [63]. Therefore, DIA1R may play a role in microglia and/or in neurovascular development, which can underlie its implication in autism and mental retardation disorders. We are currently investigating DIA1R potential function using overexpression and loss-of-function approaches in different vertebrate models.

## Figures and Tables

**Table 1:** List of selected genes upregulated in yolk sac hemangioblasts. Genes without a known function in hematopoiesis or vasculogenesis are highlighted in gray ^a^.

FC ^b^	Gene Symbol	Gene Name	Gene ID	Molecular Function	Biological Function	Expression in Early Embryos
**+5.1**	*SLC15A1*	Solute carrier family 15, member 1	378789	Membrane transporter	Oligopeptide transport	-
**+4.2**	*C1HXorf36* (*DIA1R*)	Chromosome 1 open reading frame, human CXorf36 (Deleted in Autism 1 Related)	418555	-	-	(this study)
**+4.19**	*SLC32A1*	Solute carrier family 32, member 1	419167	Vesicular transporter	GABA vesicular transporter	-
**+4.15**	*SOX7*	SRY (sex determining region Y)-box 7	771337	Transcription factor	Vasculogenesis and hematopoiesis	Angioblasts
**+3.73**	*LMO2*	LIM domain only 2	374129	Transcription factor	Hematopoiesis	Hematopoietic progenitors
**+3.7**	*CTSG*	Cathepsin G	426049	Serine protease	Tissue remodeling	Myeloid progenitors
**+3.62**	*TAL1* (*SCL*)	T-cell acute lymphocytic leukemia 1 (stem cell leukemia)	396298	Transcription factor	Hematopoiesis	Hematopoietic progenitors
**+3.51**	*RUNX1*	Runt-related transcription factor 1	396152	Transcription factor	Hematopoiesis	Blood islands
**+3.44**	*EGR1*	Early growth response 1	373931	Transcription factor	HSPC proliferation	Vasculogenic mesoderm
**+3.2**	*MRC1*	Mannose receptor C-type 1	420516	Membrane receptor	Endocytosis	Blood islands
**+3.17**	*KLHL6*	Kelch-like 6	424762	Transcription factor	Lymphocyte differentiation	-
**+3.3**	*SPI1* (*PU.1*)	Spleen focus forming virus (SFFV) proviral integration oncogene spi1	395879	Transcription factor	Hematopoiesis	Hematopoietic progenitors
**+3.07**	*RhoGap6*	Similar to Rho-GTPase-activating protein 6 (LOC422284 locus)	422284	Cytoskeleton regulator	Actin remodeling	-
**+2.77**	*LHX8*	LIM homeobox 8	424721	Transcription factor	Neurogenesis	Blood islands
**+2.71**	*FLT1* (*VEGFR1*)	Fms-related tyrosine kinase 1 (vascular endothelial growth factor receptor 1)	374100	Receptor tyrosine kinase	Vasculogenesis/Angiogenesis	Hemangioblasts and endothelial cells
**+2.63**	*SOX18*	SRY (sex determining region Y)-box 18	374200	Transcription factor	Vasculogenesis	Blood islands
**+2.49**	*CDH5*	Cadherin 5, type 2, VE-cadherin (vascular epithelium)	374068	Cell adhesion molecule	Vasculogenesis/Angiogenesis	Endothelial cells
**+2.39**	*CD34*	Hematopoietic progenitor cell antigen CD34	419856	Cell surface antigen	-	Hematopoietic progenitors
**+2.29**	*FLT4* (*VEGFR3*)	Fms-related tyrosine kinase 4 (vascular endothelial growth factor receptor 3)	395742	Receptor tyrosine kinase	Angiogenesis	Blood islands and endothelial cells
**+2.23**	*CITED4*	Cbp/p300-interacting transactivator, with Glu/Asp-rich carboxy-terminal domain, 4	395465	Transcription regulator	in vitro cardiogenesis	Blood islands
**+2.2**	*PITX1*	Paired-like homeodomain 1	374201	Transcription factor	Pituitary and hindlimb development	Posterior extraembryonic mesoderm
**+2.09**	*Fli1*	Friend leukemia virus integration 1 gene	419723	Transcription factor	Vasculogenesis and hematopoiesis	Endothelial and erythroid progenitors
**+1.96**	*HHEX*	Hematopoietically expressed homeobox	396182	Transcription factor	Vasculogenesis and hematopoiesis	Blood islands

^a^ Gene function and expression patterns were obtained from the literature and from ZFIN (http://zfin.org), GEISHA (http://geisha.arizona.edu/geisha) and EMAGE (http://www.emouseatlas.org). ^b^ FC: fold-change (lower bound).
